# Cognitive Impairments and Rehabilitation in Individuals with at-Risk Mental State for Psychosis

**DOI:** 10.3390/jpm13060952

**Published:** 2023-06-05

**Authors:** Takahiro Nemoto

**Affiliations:** 1 Department of Neuropsychiatry, Toho University Faculty of Medicine, 6-11-1 Omori-nishi, Ota-ku, Tokyo 143-8541, Japan; takahiro.nemoto@med.toho-u.ac.jp; Tel.: +81-3-3762-4151; Fax: +81-3-5471-5774; 2 Department of Psychiatry and Implementation Science, Toho University Faculty of Medicine, 6-11-1 Omori-nishi, Ota-ku, Tokyo 143-8541, Japan

## 1. Early Intervention in Psychiatry

In recent years, importance has been attached to early intervention in the field of healthcare, in general. In the psychiatric field, much effort has been focused on early detection and treatment of various psychotic disorders, including schizophrenia. It is presumed that intervention in the early stages of a disease could prevent functional decline and result in definitive improvement, thereby increasing the possibility of “recovery”. Furthermore, it is expected that early intervention can prevent psychiatric diseases from becoming overt, and vigorous research has been pursued on early intervention methods.

## 2. Early Stages of Psychotic Diseases

Psychotic diseases in the early stages are called early psychosis, and this includes first-episode psychosis (FEP) and the at-risk mental state (ARMS), which is associated with a high risk of overt onset of psychosis. Schizophrenia takes the central position among psychotic diseases.

In patients with first-episode schizophrenia, biological and psychological changes, which are common in the chronic stage of the disease, are already present. A cross-sectional study has shown significant and progressive volume decreases in more than one cerebral region in patients with first-episode schizophrenia as compared with healthy individuals [1]. In addition, cognitive impairment involving multiple domains is already present in patients with first-episode schizophrenia, although the degree of impairment is milder than that in patients in the chronic stage of the disease [2]. In other words, progressive biological and psychological deterioration is already present during FEP. In the chronic stage, such deterioration follows a stable course. These findings indicate the importance of reducing the duration of untreated psychosis (DUP) [3].

Attention to the early stage of disease could include FEP to further along the course of the disease, and intervention at the stage of the prodrome prior to overt disease onset is now discussed actively. Evaluation and diagnosis of this prodromal state or ARMS, i.e., a state that entails a high risk of development of overt psychiatric illness, are performed by prospective recognition of this state. A meta-analysis on transition from ARMS to psychosis showed that the cumulative onset rate of overt disease was 35.8% [4].

Individuals with ARMS often have social problems related to attending school or going to work, and in parallel with these problems, general psychiatric manifestations and weak psychotic symptoms gradually become manifest. Some patients with ARMS seek advice from medical or health organizations to obtain support and treatment for their problems and symptoms. These patients are called “help-seeking individuals”, and patients with ARMS who seek support should be the targets of early intervention from the ethical viewpoint as well.

## 3. The Process of Formation of Cognitive Impairments in ARMS

Studies using magnetic resonance imaging (MRI) have revealed that patients with ARMS already show decreases in the volume of cerebral gray matter at the time of diagnosis, in contrast to the case in healthy individuals, and these volume decreases continue to progress until the disease becomes overt. In addition, a comparison of the brain images obtained at the baseline between ARMS patients who eventually show/do not show overt disease revealed that there was already significant volume loss of the parahippocampal gyrus at the baseline in the former group of patients [5]. Decreased integrity of the white matter on diffusion tensor imaging (DTI) has also been reported [6].

A meta-analysis has shown slight to moderate decreases in multiple domains of neurocognition, together with decreases in social cognition, in ARMS patients as compared with healthy individuals [7]. When the data were compared between those who eventually developed/did not develop overt, neurocognitive impairments at the baseline were more severe in the patients who eventually developed overt disease versus those who did not [7]. Thus, it has become evident that anatomical and functional changes in the brain progress during the course of the disease, right from the prodromal phase to overt disease onset.

A systematic review of the differences in the degree of cognitive impairments between ARMS patients and healthy individuals or patients with FEP [8] showed that all of the studies included in the review (24 studies) reported significantly poorer cognition in patients with ARMS than in healthy individuals, and two-thirds of the reports examined (nine out of fourteen studies) showed that the degree of cognitive impairments were milder in ARMS patients than in patients with FEP. This review (all 10 studies examined) also showed that social cognition was significantly poorer in ARMS patients than in healthy individuals, but that the degree of decrease in social cognition was equivalent to that in patients with FEP, according to three of the four studies examined. Thus, there is a distinct decrease in neurocognitive functions and social cognition even in the ARMS phase, and a comparison with FEP suggests the problem of impairment of social cognition becomes apparent earlier, in the ARMS phase.

## 4. Cognitive Rehabilitation for ARMS

In patients with schizophrenia, it has been shown that cognitive impairments, rather than positive or negative symptoms, are the major determinant of the social functioning of the patients. This is the rationale for using cognitive rehabilitation as an early intervention in these patients, with the aim of improving the long-term outcomes [9]. In recent years, since the involvement of social cognition and also the greater degree of involvement of social cognition, rather than basic cognitive functions, has come to be recognized, and rehabilitation intervention for social cognition by means of social cognition and interaction training (SCIT) has been implemented [10]. Based on these findings about schizophrenia, the feasibility and efficacy of cognitive rehabilitation for ARMS have come to be evaluated.

A systematic review on the efficacy of cognitive remediation in ARMS patients [11] included six studies that met the inclusion criteria [12,13,14,15,16,17]. None of the six studies used any compensatory approaches involving environmental adjustment, but used restorative approaches that take into consideration plasticity and changes in the brain function, by means of drill learning using a computer. Although cognitive remediation was regarded as a part of comprehensive treatment in two studies, individual cognitive function training was implemented in all six studies, without employing group work intended for extrapolation to daily living. While the effect of cognitive remediation on the cognitive function was not evaluated in one of the studies, four of the five remaining studies reported improvement in attention, processing speed, and memory function following the intervention. Four of the studies examined the effect of the intervention on social functioning. Two studies reported improvement, while the remaining two reported no distinct effect of the intervention on social functioning. None of the studies which examined this aspect showed significant improvement in the mental symptoms following the intervention. One study examined the transition from ARMS to overt onset of psychosis and reported that the intervention exerted a good preventive effect at 12 and 24 months, as compared with supportive therapy. However, since cognitive remediation is a part of comprehensive treatment, including cognitive behavioral therapy (CBT) and family psychological education, it should be noted that the preventive effect cannot be attributed to cognitive remediation alone.

On the other hand, the recent randomized clinical trial, named FOCUS, in which individuals with ARMS aged 18–40 years were randomly assigned to treatment as usual (TAU) or TAU plus cognitive remediation that also targeted social cognition did not reveal between-group differences on mail outcomes [18,19]. In this study, it appears that the participants did not adhere well to the protocol. Empirically, it is not easy to introduce young people to treatment and maintain it with good compliance. This may be one of the biggest barriers to treating individuals with ARMS.

Currently, there are only a few reports on the effects of rehabilitation targeting social cognition in ARMS patients.

## 5. Possibility of Cognitive Rehabilitation

Although cognitive rehabilitation in ARMS patients has not yet been sufficiently studied, its effects on the cognitive functions and social functioning is considered to be promising, at least to some extent. Based on the documentation of successful prevention of volume decreases in the left hippocampus, parahippocampal gyrus, fusiform gyrus, etc., by cognitive rehabilitation in the early stage of schizophrenia [20], a favorable effect may also be expected on the progression of changes in the brain structure in the ARMS phase. However, in the FOCUS study, cognitive remediation did not improve global or regional white matter organization in ARMS individuals [21], and it seems that favorable results have not been obtained so far.

On the other hand, the involvement of cognitive functions in social functioning is less in ARMS than in schizophrenia, with negative symptoms playing a more dominant role [22]. Therefore, in ARMS patients, an aggressive approach to not only cognitive function rehabilitation, but also other factors, is necessary. In the chronic stage of schizophrenia, both cognitive functions and social functioning are relatively stable, with no particular changes in the brain morphology occurring during this phase. In contrast, brain morphology and brain functions show great changes in the ARMS phase, which serves as a good rationale for using multiple therapeutic targets.

Cornblatt et al., [23], referred to the importance of “CASIS” in ARMS, in addition to positive symptoms, playing a decisive role in whether a patient develops overt psychosis or not. In “CASIS”, C stands for cognitive deficits, A for affective disturbances, SI for social isolation, and S for school failure. These disorders are reported to be risk factors for the development of overt psychosis and are, therefore, important therapeutic targets. We have been investigating social isolation, paying particular attention to the involvement of social anxiety [24,25,26]. While social anxiety in schizophrenia can occur as a psychosocial reaction related to stigmas after overt onset of psychosis, it has been pointed out that social anxiety also exists in the premorbid or prodromal phase, serving as a risk factor for the onset of overt psychosis. In addition, a birth cohort study of schizophrenia has indicated social anxiety, isolated play, and lack of confidence as characteristic features of social difficulties in schoolage children.

## 6. Future Perspectives of Treatment for ARMS

For patients with FEP, antipsychotic drug therapy should be initiated as soon as possible. In contrast, in patients with ARMS, use of antipsychotic drugs is recommended only when there is evidence of rapid aggravation of the disease [27]. Otherwise, non-pharmacologic therapy is the mainstay of treatment for these patients, and there is much expectation from cognitive rehabilitation as a type of non-pharmacologic therapy. Further accumulation of findings about this intervention is awaited. Training on social cognition has hardly been addressed.

In a study conducted by Chen et al. [28], FEP patients who were not aware of recovery were asked about the reason, and “decreased cognitive function” and “need to continue medication” were found to be the predominant reasons. Although the cognitive function disorder in ARMS patients is milder than that in FEP patients, patients with ARMS are strongly aware of decreased cognitive function because of maintained self-insight. Therefore, it is evident from daily clinical practice that improvement of cognitive function ranks relatively high among the interventions that ARMS patients can expect from healthcare providers. It is desirable for cognitive rehabilitation to be implemented more widely. To this end, however, adjusting the difficulty level of training in accordance with the need of the patients and improving the environment of its implementation are necessary. Moreover, as mentioned above, it is indispensable to employ more comprehensive and integrated approaches for ARMS patients as compared with FEP patients.

With the greater orientation of efforts towards mental health maintenance and early intervention towards the local community (universal prevention) [29,30], there is urgent need to develop a specific treatment and support package for ARMS, giving due consideration to and making use of the healthcare system and social resources, as well as to verify its efficacy.
